# Identification of Methylation-Driven, Differentially Expressed *STXBP6* as a Novel Biomarker in Lung Adenocarcinoma

**DOI:** 10.1038/srep42573

**Published:** 2017-02-15

**Authors:** Govinda Lenka, Mong-Hsun Tsai, Hsin-Chieh Lin, Jen-Hao Hsiao, Yi-Ching Lee, Tzu-Pin Lu, Jang-Ming Lee, Chung-Ping Hsu, Liang-Chuan Lai, Eric Y. Chuang

**Affiliations:** 1 Graduate Institute of Physiology, National Taiwan University, Taipei, Taiwan; 2 Institute of Biotechnology, National Taiwan University, Taipei, Taiwan; 3 Bioinformatics and Biostatistics Core, Center of Genomic Medicine, National Taiwan University, Taipei, Taiwan; 4 Graduate Institute of Biomedical Electronics and Bioinformatics, National Taiwan University, Taipei, Taiwan; 5 Institute of Cellular and Organismic Biology, Academia Sinica, Taipei, Taiwan; 6 Department of Public Health, Institute of Epidemiology and Preventive Medicine, National Taiwan University, Taipei, Taiwan; 7 Department of Surgery, National Taiwan University Hospital, Taipei, Taiwan; 8 Division of Thoracic Surgery, Taichung Veterans General Hospital, Taichung, Taiwan; 9 School of Chinese Medicine, China Medical University, Taichung, Taiwan

## Abstract

DNA methylation is an essential epigenetic marker associated with the silencing of gene expression. Although various genome-wide studies revealed aberrantly methylated gene targets as molecular biomarkers for early detection, the survival rate of lung cancer patients is still poor. In order to identify methylation-driven biomarkers, genome-wide changes in DNA methylation and differential expression in 32 pairs of lung adenocarcinoma and adjacent normal lung tissue in non-smoking women were examined. This concurrent analysis identified 21 negatively correlated probes (r ≤ −0.5), corresponding to 17 genes. Examining the endogenous expression in lung cancer cell lines, five of the genes were found to be significantly down-regulated. Furthermore, in tumor cells alone, 5-aza-2′-deoxycytidine treatment increased the expression levels of *STXBP6* in a dose dependent manner and pyrosequencing showed higher percentage of methylation in *STXBP6* promoter. Functional analysis revealed that overexpressed *STXBP6* in A549 and H1299 cells significantly decreased cell proliferation, colony formation, and migration, and increased apoptosis. Finally, significantly lower survival rates (*P* < 0.05) were observed when expression levels of *STXBP6* were low. Our results provide a basis for the genetic etiology of lung adenocarcinoma by demonstrating the possible role of hypermethylation of *STXBP6* in poor clinical outcomes in lung cancer patients.

Lung carcinoma is one of the most commonly diagnosed cancer types, and it is characterized by poor survival rates. According to recent global cancer statistics, it accounts for 18% of all cancer-related deaths worldwide^1^. Despite decades of research efforts to improve the clinical outcomes of lung cancer patients, the overall survival rates remain dismal. The mortality rates of lung cancer were the highest in Taiwan^2^,^3^.

A wide variety of risk factors, such as genetic, epigenetic, and environmental factors, may cause lung cancer. Nearly 70–90% of lung cancers in Western countries are caused due to cigarette smoking, but only 7% of female lung cancer cases are associated with smoking^4^,^5^,^6^. Furthermore, adenocarcinoma is much more common than other subtypes of non-small cell lung carcinoma seen in non-smokers. These statistics emphasize the necessity of a better understanding of the molecular mechanisms that mediate the development of lung cancer in non-smoking female patients.

Advanced high-throughput technologies have had an important role in identifying the genetic abnormalities that drive the development and growth of various cancers^7^,^8^. In addition to genetic changes, epigenetic changes, such as hyper- or hypomethylation, lead to the aberrant expression of tumor suppressor genes or oncogenes^9^. Hypermethylation within the promoter region was responsible for the inactivation of approximately half of the classical tumor suppressor genes^10^,^11^. Tumor suppressor genes undergoing aberrant hypermethylation were expressed in a non-random, tumor-specific pattern in many cancer types^12^. Therefore, epigenetically disrupted gene expression was able to alter various cancer-related processes, such as cell cycle checkpoints, cell proliferation, apoptosis, signal transduction, regulation of transcription factors, cell adhesion, and angiogenesis^13^,^14^. Also, various molecular genetics investigations revealed the impact of methylation on either resistance or sensitivity to chemotherapy or radiation^15^,^16^,^17^,^18^,^19^.

The possible role of DNA methylation in lung cancer was identified based on analysis of sputum^20^ and in prognosis of early-stage lung cancer^21^,^22^. Unlike other genetic alterations, methylation-based epigenetic modification is an inherently reversible change, due to which it has gained much attention as an active target of drug development. Therefore, over the past few decades, several research groups have been focused on finding the epigenetic markers (e.g., *APC*^23^ and *SHOX2*^24^,^25^) or a group of gene set, such as (*APC, RASSF1A, CDH13, KLK10* and *DLEC1*)^26^ and (*AGTR1, GALR1, SLC5A8, ZMYND10* and *NTSR1*)^27^, for detection or diagnosis of lung cancer. However, the role of methylation in the tumorigenesis of lung adenocarcinoma and association with prognosis in Taiwan remains largely unknown. For this reason, we performed an integrated analysis of gene expression and DNA methylation status to find novel epigenetic markers of lung cancer. With this approach, we identified *STXBP6*, whose expression was significantly repressed by methylation, affected cellular function in cancer cell lines, and was associated with overall survival.

Syntaxin binding protein 6, encoded by *STXBP6*, was initially identified in regulating the formation of the SNARE complex^28^ and cytogenesis^29^. The regulatory role of *STXBP6* in exocytosis and fusion pore stability was performed by both syntaxin-dependent and syntaxin-independent mechanisms^30^. It has been reported to be associated with many diseases, such as diabetes^31^, autism^32^,^33^, and systemic lupus erythematosus^34^. However, there are no studies revealing its biological role in association with lung cancer and its epigenetic regulation.

Therefore, in this study we explored the epigenetic inactivation of *STXBP6* expression using lung adenocarcinoma patients. Pyrosequencing analysis using *in vitro* cellular models revealed the specific CpG sites that are responsible for the hypermethylation of *STXBP6*. Functional analysis revealed the tumor-suppressive role of *STXBP6* in *in vitro* lung cancer cellular models. Finally, poor survival rates were observed in patients with low expression levels of *STXBP6*. Thus, methylation-driven, differentially expressed *STXBP6* may be used as a novel biomarker to predict clinical outcomes of lung adenocarcinoma patients.

## Results

### Differential expression and methylation profiling in lung adenocarcinoma

**Figure 1:**
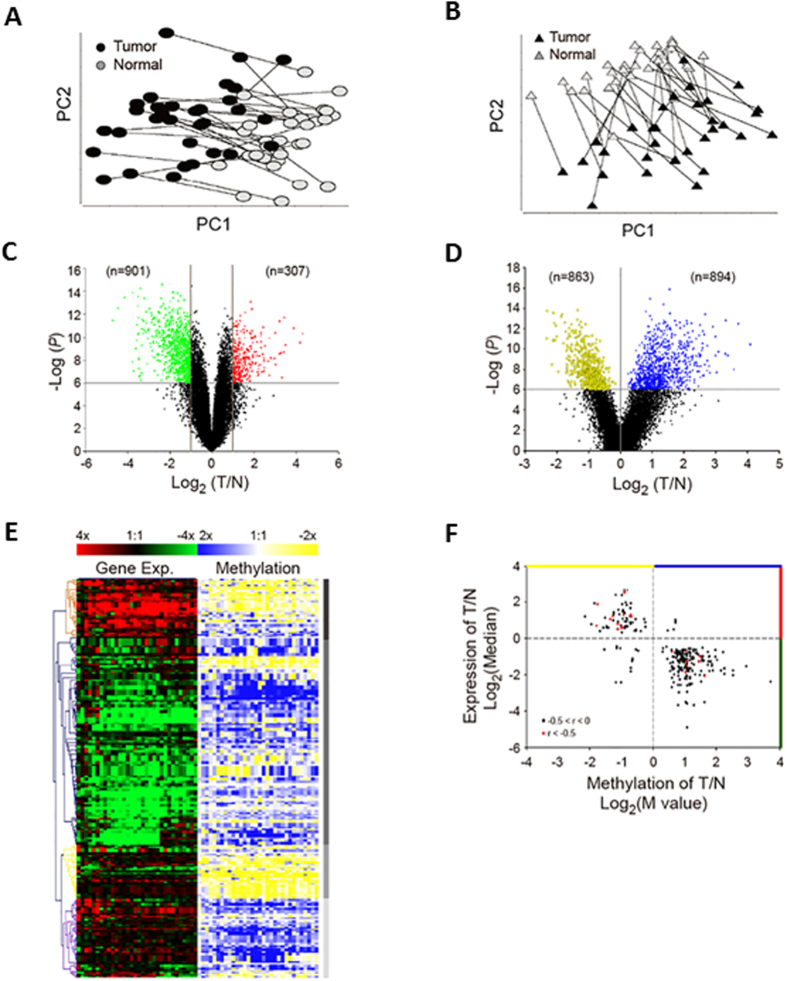
Identification of differentially expressed genes driven by methylation in non-smoking women with lung adenocarcinoma. (**A**) Principal component analysis of probes with differential gene expression in tumor and normal tissues. Differentially expressed probes were identified by paired t tests (*P* ≤ 10^−6^) and fold change (log_2_ ≥ 1 or ≤ −1) in tumor/normal lung tissues. Connecting lines indicate paired samples from the same individual. (**B**) Principal component analysis of probes with differential methylation status in tumor and normal tissues. The intensities of methylated probes versus unmethylated probes were converted to M values and examined by paired t tests (*P* ≤ 10^−6^). (**C**) Volcano plot of differentially expressed genes. Green dots indicate down-regulated probes (n = 901) and red dots denote up-regulated probes (n = 307). (**D**) Volcano plot of probes with differential DNA methylation status. Yellow dots denote hypomethylated probes (n = 863) and blue dots indicate hypermethylated probes (n = 894). (**E**) Heat map of the probes (n = 273) showing negative correlation (r < 0) between differential expression and methylation status. Hierarchical cluster analysis was performed using Pearson correlation distance. (**F**) Starburst plot of probes showing negative correlation between gene expression (y-axis) and DNA methylation (x-axis).

In this genome-wide study, we sought to identify the genes whose expression was differentially regulated by DNA methylation in lung cancer cells. Genome-wide expression (41,789 probes) and DNA methylation profiling (27,578 probes) in 32 pairs of tumor and adjacent normal tissues were analyzed in non-smoking women with lung adenocarcinoma (Table S2). The average age of patients was 62 years old and 78% of them were in Stage I or II. To visualize the distribution of tumor and normal samples based on expression or methylation levels, principal component analyses was executed using differentially expressed probes of gene expression (Fig. 1A) and DNA methylation (Fig. 1B). Black dots denote tumor tissues, gray dots denote normal tissues, and each line indicates the paired samples from the same individual. Each dot represents the expression (Fig. 1A) or methylation values (Fig. 1B) of the significant probes that were summarized at the first two principal component coordinates. The results showed the distinct separation of tumor samples from their corresponding normal samples, indicating distinct patterns of gene expression and methylation levels in normal and tumor tissues.

Differential expression of probes was filtered by fold change ( ≥ 2-fold) and statistical significance (*P* ≤ 10^−6^) in pairs of tumor and normal lung tissues. As shown in Fig. 1C, 901 probes were found to be down-regulated (log_2_ ≤ −1), whereas 307 probes were up-regulated (log_2_ ≥ 1). After the intensities of methylated probes were converted to M values and examined by paired-t tests (*P* ≤ 10^−6^), 863 probes were found to be hypomethylated, whereas 894 probes were hypermethylated (Fig. 1D). To correlate genome-wide methylation changes with concomitant changes in expression, we integrated the gene expression and DNA methylation probe pairs. In this analysis, we identified 273 negatively correlated probe pairs from 50,948 combined gene expression and methylation probe pairs, meaning that gene expression and methylation changed in opposite directions. Heat maps were used to represent the negative correlation (r < 0) between gene expression and methylation status for these 273 probe pairs (Fig. 1E). Hierarchical cluster analysis identified a distinct cluster for the up-regulated (red) and hypomethylated (yellow) genes. Similarly, down-regulated (green) genes with hypermethylation (blue) status were represented as another cluster. The results depicted in the heat map indicate a clear negative correlation between DNA methylation and genes expression profiles in tumor samples (Fig. 1E; Supplementary Dataset 1).

In order to screen candidate genes for validation, we then narrowed down the probes by increasing the stringency of negative correlation (r ≤ −0.5) between gene expression and DNA methylation (Fig. 1F). As shown in Table 1, 21 probes corresponding to 17 genes met this criterion. These results emphasize that abnormal DNA methylation might play a role in the regulation of 17 genes showing significant differential expression in lung adenocarcinoma.

### Identification of methylation-driven down-regulated genes in lung cancer cell lines

**Figure 2:**
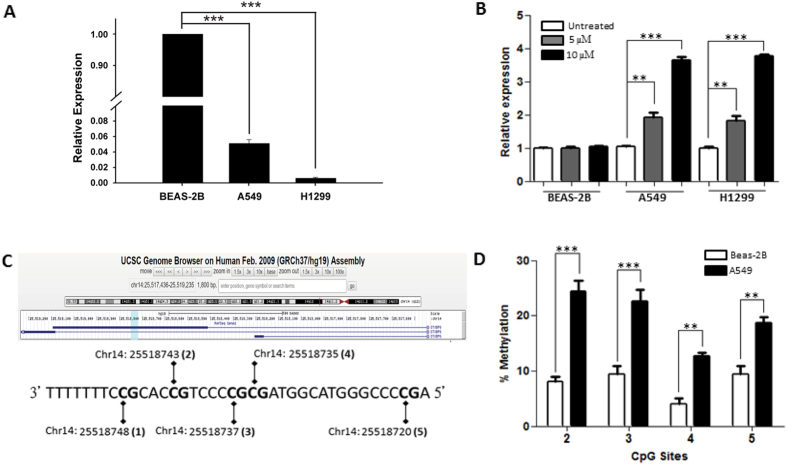
Validation of *STXBP6* regulated by methylation in lung cancer cells. (**A**) Endogenous levels of *STXBP6* in lung cancer cells (A549 and H1299) and BEAS-2B normal control cells. The mRNAs were measured by quantitative RT-PCR. *GAPDH* was the internal control. Relative expression levels were normalized against BEAS-2B cells. Bars represent the means ± SDs of 3 independent experiments. (**B**) Relative expression of *STXBP6* in cells treated with 5-aza-2-deoxycytidine (5-aza). Cells were treated with 5 and 10 μM of methylation inhibitor 5-aza for 3 days. Relative expression levels of *STXBP6* were normalized against the untreated group of each cell line. (**C**) Scheme of CpG sites in the 5′-untranslated region of *STXBP6* for pyrosequencing. Top: CpG sites for pyrosequencing were highlighted in light blue area. Bottom: CpG sites were numbered as shown in parentheses according to genome assembly version (GRCh37/hg19). (**D**) Quantification of DNA methylation of *STXBP6* using pyrosequencing analysis. Genomic DNA was extracted from A549 and BEAS-2B cells and treated with bisulfide to examine the methylation percentage of CpG sites by pyrosequencing. **P ≤ 0.001, ***P ≤ 0.0001.

To validate differentially expressed genes driven by methylation in non-smoking women with lung adenocarcinoma and select candidate genes for functional analysis, endogenous expression levels of the 17 genes were examined in lung cancer cell lines (A549 and H1299). Five genes, including *IL11RA, GSTM*5, *STXBP6, RHOJ*, and *PECAM1*, were significantly down-regulated (*P* ≤ 0.0001) in A549 and H1299 cells as compared to normal BEAS-2B cells (Figs 2A and S1A–D).

To validate the role of methylation in the regulation of the expression of these 5 genes, we treated A549, H1299, and BEAS-2B cell lines with 5-aza. Interestingly, only *STXBP6*, which expression was significantly up-regulated in a dose-dependent manner, was found when the A549 and H1299 cell lines were treated with 5-aza (Fig. 2B). In contrast, non-significant differences were observed in BEAS-2B normal cells (Fig. 2B). Furthermore, pyrosequencing analysis was performed to identify the specific CpG sites of methylation in *STXBP6*. Five CpG sites in the 5′ untranslated region of *STXBP6* were examined (Fig. 2C). Four of the five CpG sites (the CpG site at Chr14:25518748 was undetected) showed a significantly higher methylation percentage in A549 cells compared to normal BEAS-2B cells (Fig. 2D). Lastly, as shown in Table 2, the expression levels of *STXBP6* were significantly down-regulated in adenocarcinoma samples in eight data set. Also, the methylation levels of *STXBP6* were significantly hypermethylated in six data set (Table 2). While datasets containing both expression and methylation data, the converse relationship between methylation and gene expression of *STXBP6* was not only observed in our data set (GSE19804/GSE49996) but also in three publicly available data set (Table 2; Supplementary Dataset 2). These findings suggested a possible role of methylation in down-regulating the expression of *STXBP6* in lung cancer cells.

### Functional investigation of *STXBP6* in lung cancer cell lines

**Figure 3:**
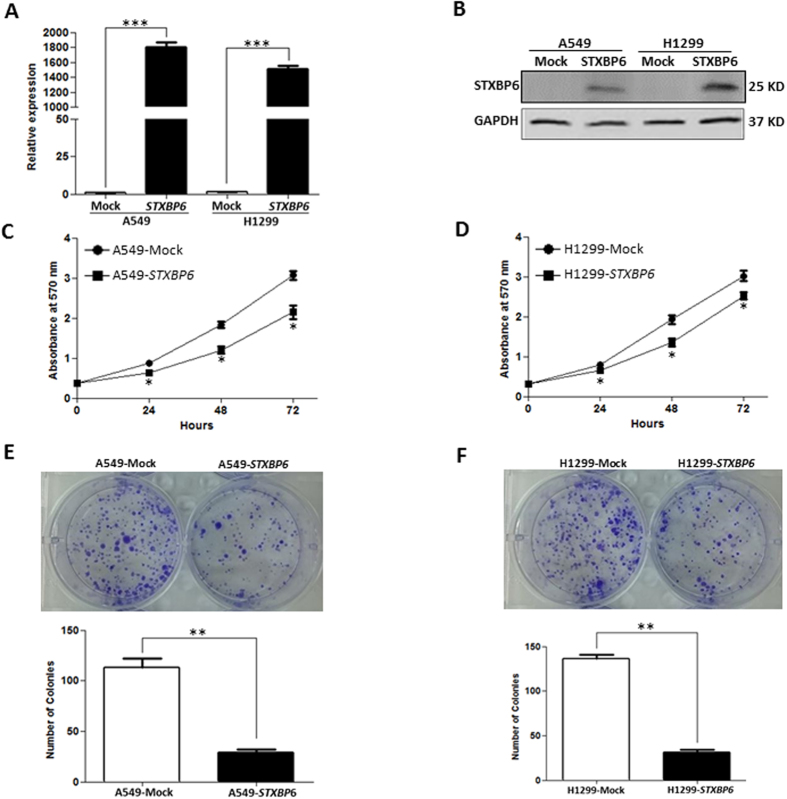
*STXBP6* suppresses cell proliferation and colony formation in lung cancer cells. (**A**) Relative expression levels of *STXBP6* in A549 and H1299 cells overexpressing *STXBP6* by quantitative RT-PCR. *GAPDH* was the internal control. Bars represent the means ± SDs of 3 independent experiments. (**B**) Western blot analysis of STXBP6 in A549 and H1299 cells overexpressing *STXBP6*. GAPDH was used as the loading control. (**C**) MTT assays of A549 cells overexpressing *STXBP6*. Proliferation assays were executed by adding MTT at different time points after seeding 4,000 cells/well. (**D**) MTT assays of H1299 cells overexpressing *STXBP6*. (**E**) Colony formation assays of A549 cells overexpressing *STXBP6*. A representative image is shown above and a quantitative graph below. (**F**) Colony formation assays of H1299 cells overexpressing *STXBP6*. A representative image is shown above and a quantitative graph below. *P < 0. 05, **P < 0. 001, ***P < 0.0001.

Since *STXBP6* was epigenetically down-regulated in lung cancer cells, we investigated the functional roles of *STXBP6* by transiently transfecting a *STXBP6* expression plasmid into A549 and H1299 cells. As shown in Fig. 3A, the mRNA levels of *STXBP6* in A549 and H1299 cells after transfection were significantly increased (*P* ≤ 0.0001). Western blot analysis also validated the increased protein amounts of *STXBP6* in both A549 and H1299 cells upon transfection of *STXBP6* plasmid (Fig. 3B).

**Figure 4:**
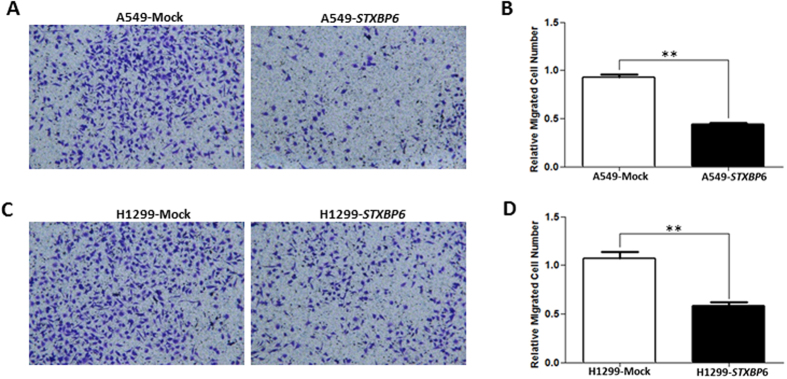
*STXBP6* decreases cell migration in lung cancer cells. (**A**) Representative image of the bottom surface of a transwell migration assay in A549 cells. (**B**) Quantification of migrated cells in (**A**). (**C**) Representative image of the bottom surface of a transwell migration assay in H1299 cells. (**D**) Quantification of migrated cells in (**C**). The absorbance values of migratory cells were normalized against a mock transfection group. Bars represent the means ± SDs of 3 independent experiments. **P < 0.001.

After successfully overexpressing *STXBP6* in lung cancer cells, we first examined the effect of *STXBP6* on cell growth by MTT assays. The results showed a significant decrement in proliferation in both A549 and H1299 cells overexpressing *STXBP6 (P* ≤ 0.05) (Fig. 3C,D). Furthermore, *STXBP6* overexpression markedly reduced colony formation in both A549 and H1299 cells (Fig. 3E,F). Next, the role of *STXBP6* in migration of lung cancer cell lines was investigated by transwell migration assays. The results revealed that *STXBP6* significantly suppressed the migration abilities of both A549 and H1299 cells (*P* ≤ 0.001) (Fig. 4A–D).

**Figure 5:**
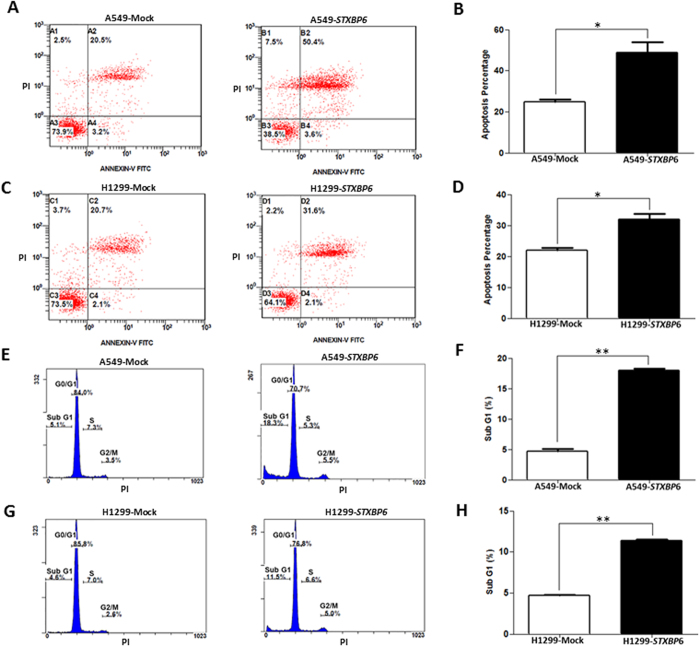
*STXBP6* increases apoptosis in lung cancer cells. (**A**) A representative diagram of annexin V-FITC and propidium iodide (PI) staining assays in A549 cells overexpressing *STXBP6*. (**B**) Quantification of annexin V results in (**A**). The percentage of apoptotic cells was derived from the sum of the percentages of late and early apoptotic cells. (**C**) A representative diagram of annexin V-FITC and PI staining assays in H1299 cells overexpressing *STXBP6*. (**D**) Quantification of annexin V-FITC results in (**C**). (**E**) Flow cytometry analysis for apoptosis in A549 cells overexpressing *STXBP6*. (**F**) Quantification of apoptotic cells in (**E**). The percentage of cells in sub-G1 phase was used to identify the apoptotic cells by PI staining. Bars represent the means ± SDs of 3 independent experiments. (**G**) Flow cytometry analysis for apoptosis in H1299 cells overexpressing *STXBP6*. (**H**) Quantification of apoptotic cells in (**G**). *P < 0. 05, **P < 0. 001.

To evaluate the possible significance of *STXBP6* expression in the modulation of apoptosis, annexin V-FITC and PI staining were carried out. The results showed a noticeable increment in apoptotic percentage in both A549 and H1299 cells overexpressing *STXBP6 (P* ≤ 0.05) (Fig. 5A–D). Furthermore, cell cycle analysis was performed on day 3 of transfection with the *STXBP6* plasmid. The percentage of apoptotic cells in G1 phase was significantly increased in both A549 and H1299 cells overexpressing *STXBP6 (P* ≤ 0.001) (Fig. 5E–H). These results demonstrated the suppressive effects of *STXBP6* on lung carcinogenesis.

### Expression levels of *STXBP6* in tumor tissues correlate with patients’ overall survival

**Figure 6:**
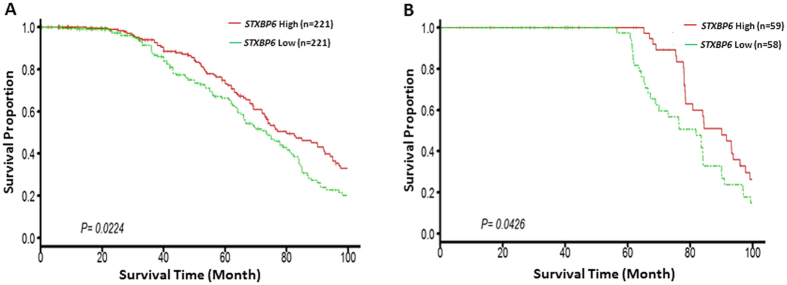
Down-regulation of *STXBP6* in lung cancer tissues was associated with poor overall survival. Patients were divided into “high expression” or “low expression” groups based on the median value of all samples. Kaplan–Meier analysis was used to examine overall survival in relation to the expression values of *STXBP6* in adenocarcinoma patients from (**A**) Shedden’s study^35^ and (**B**) Tomida’s study^36^. *P*-values were calculated by a log-rank test.

Lastly, Kaplan–Meier analysis was used to examine overall survival in relation to the expression values of *STXBP6* in adenocarcinoma patients from Shedden’s study^35^ (Fig. 6A) and Tomida’s study^36^ (Fig. 6B). Patients were divided into “high expression” or “low expression” groups based on the median value of all samples. The results showed that patients with lower expression of *STXBP6* had poorer survival than those with high expression (Fig. 6A,B). These findings indicate that epigenetic changes in *STXBP6* may be useful for predicting the prognosis of patients with lung adenocarcinoma.

## Discussion

In this study, we sought to uncover epigenetic-based molecular targets by analyzing the association between genome-wide DNA methylation and gene expression patterns in tumor and adjacent normal tissues from non-smoking Taiwanese female lung adenocarcinoma patients. First, differential expression levels and methylation status between tumor and normal tissues were identified using both gene expression and methylation microarrays. Second, selected genes with negative correlations between expression levels and methylation status were then validated by examining their endogenous levels in lung cancer cell lines. Third, pyrosequencing and 5-aza-2′-deoxycytidine treatment showed the regulatory role of methylation of *STXBP6* in tumor cells. Fourth, functional analysis revealed that *STXBP6* suppressed tumor growth in lung cancer cell lines. Finally, lower expression of *STXBP6* was found to be associated with poor clinical outcomes in lung cancer patients.

Because of the development of targeted therapy resistance or the absence of targetable mutations in lung cancer patients, developing alternative therapeutic strategies for lung cancer in early diagnosis, prognosis prediction, and treatment are urgent and important. Epigenetics approaches, including DNA methylation, histone modification, and miRNA regulation, may solve these problems by affecting multiple pathways that regulate major properties of the cancer cells. Among them, DNA methylation at CpG sites is the most characterized epigenetic modification described in lung cancer. Therefore, targeting DNA methylation of tumor suppressor genes or oncogenes may hold promise in lung cancer therapy.

For the last several years, more and more evidence has accumulated to emphasize the hypermethylation status of CpG islands located in the promoter regions of tumor suppressor genes^37^. Several groups have performed epigenetic analyses of methylation in types of cancer other than lung adenocarcinoma. A recent genome-wide analysis of DNA methylation and gene expression changes in lung squamous cell carcinoma identified several methylation-driven genes, including *CCDC37, CYTL1, CDO1, SLIT2, LMO3* and *SERPINB5*^38^. Another genomic analysis of idiopathic pulmonary fibrosis identified methylation–gene expression relationships within genes that were either involved in fibroproliferation or were feasible candidates in this process^39^. Suzuki *et al*. performed an integrative multi-omics analysis to understand how cancers harbor various types of aberrations at the genomic, epigenomic, and transcriptional levels^40^. Additional investigations using larger numbers of samples with varied clinical features can help to reveal novel gene targets associated with methylation changes in tumorigenesis. Further research direction may also include the timing of methylation and the difference in methylation levels between epithelial and stromal tissues. In addition, more studies should focus on finding markers for epigenetic priming agents that render lung cancer more susceptible to cytotoxic chemotherapy and immunotherapy.

Worsening lung cancer statistics (e.g., increasing global incidence), particularly in women^41^,^42^, invoke researchers to develop accurate and highly sensitive markers for the early detection of disease. Several biomarkers for the diagnosis of lung cancer have been identified^43^; however, sensitivities of these biomarkers differ for each subtype of lung cancer. Thus, it is also highly challenging to find specific biomarkers for each subtype, as the various lung cancers are known to have diverse pathological features. Hence, we set out to find suitable biomarker genes for adenocarcinoma, to distinguish it from normal samples. To meet this expectation, we processed our data using a stringent cutoff for the negative correlation between gene expression and DNA methylation in non-smoking women with lung adenocarcinoma. From the genome-wide analysis, 167 methylation-driven, differentially expressed genes (273 probes) were identified in lung adenocarcinoma. In spite of differences in clinical features of tumor samples and selection criteria, previous genome-wide methylation studies showed results similar to our study^44^,^45^. For instance, our hierarchical clustering analysis also resulted in a large cluster for most of the probes, which are hypermethylated and down-regulated. The current findings along with previous findings indicate that a larger number of genes may undergo hypermethylation in the case of lung carcinogenesis.

Interestingly, differential expression of some of the candidate genes in our study was in agreement with previous studies. For example, *CDKN3* was found to be overexpressed in hepatocellular carcinoma and to promote cell proliferation by affecting cell cycle progression^46^, and was also found be overexpressed in this study. To the best of our knowledge, this is the first study reporting the regulatory role of methylation in the control of *STXBP6* in lung cancer with different validation approaches, including microarrays and 5-aza and pyrosequencing analyses. Moreover, *STXBP6* was significantly down-regulated or hypermethylated in many public data set (Table 2)^44^,^47^,^48^,^49^,^50^. Furthermore, negative correlation between gene expression and methylation status for *STXBP6* was observed in other three publicly available datasets (Table 2)^44^,^51^, indicating the possibility of epigenetic inactivation of *STXBP6* even in different races. The possible role of methylation in the control of *STXBP6* expression was also suggested by a human cell model of breast cancer^52^,^53^. These results indicate that epigenetic silencing of *STXBP6* could occur in different cancer types.

Administration of methylation inhibitors, such as 5-aza, is one of the most commonly used strategies to uncover the role of aberrant methylation changes in gene inactivation^54^,^55^. When cells were treated with 5-aza, up-regulation of *STXBP6* was observed only in cancerous cell lines. Pyrosequencing results further identified the methylated CpG sites modulating the expression of *STXBP6* in these cell lines.

To investigate the functional roles of *STXBP6*, it was overexpressed in A549 and H1299 cancer cells. Ectopic expression of *STXBP6* resulted in slower cell proliferation, less colony formation, slower migration ability, and a greater percentage of apoptosis in lung cancer cells. These results suggested that *STXBP6* could function as a tumor suppressor, although further experiments are warranted using *in vivo* studies.

Lastly, survival analysis using two publicly available datasets^35^,^36^ indicated that the survival probability of lung cancer patients increased with higher expression of *STXBP6* in tumor tissues. This result suggested an avenue for developing a novel therapeutic regimen for treating lung cancer.

In conclusion, our results indicate that the pathogenesis of lung adenocarcinoma may result from epigenetically regulated expression levels of *STXBP6*. Before this biomarker can be translated into clinical utility, further studies using larger sample sizes will help to reveal the importance of *STXBP6* as novel potential biomarker for the prognosis of lung adenocarcinoma^56^. Multicenter studies are also needed to validate the tests and analyze the reproducibility of promising results derived from limited samples.

## Methods

### Clinical tissue samples

Thirty-two pairs of lung adenocarcinoma and adjacent normal lung tissue samples were acquired from non-smoking female patients admitted to National Taiwan University Hospital or Taichung Veterans General Hospital. Written informed consent was obtained from all subjects and/or guardians for the use of their tissue samples. Acquisition and subsequent use of all the clinical samples were in accordance with the Declaration of Helsinki, and were approved by the Institutional Review Board of National Taiwan University Hospital (IRB approval Number: 200610015 R) and the Institutional Review Board of Taichung Veterans General Hospital (IRB approval Number: C09204). Lung tissue samples were quickly immersed in RNAlater® solution (Life Technologies, Gaithersburg, MD, USA), snap-frozen in liquid nitrogen, and stored at −80 °C. Before the extraction of RNA and DNA, frozen tumor tissue blocks were sectioned.

### RNA extraction and cDNA synthesis

Total RNA from sectioned tissue samples was isolated using TRIzol reagent (Life Technologies, Gaithersburg, MD, USA) and purified with the RNeasy mini kit (Qiagen, Hilden, Germany) according to the manufacturer’s instructions. RNA integrity was confirmed by agarose gel electrophoresis and the Agilent 2100 Bioanalyzer RNA 6000 LabChip kit (Agilent Technologies, Santa Clara, CA, USA). The purified total RNAs were then used as templates to synthesize the labeled double-stranded cDNA and cRNA according to the Affymetrix standard synthesis protocols.

### Genomic DNA isolation, bisulfite treatment, and methylation profiling

Genomic DNA from tumor and adjacent normal tissue samples was extracted using TRIzol reagent (Life Technologies, Gaithersburg, MD, USA). The DNA was then subjected to bisulfite conversion using an EZ DNA methylation kit (Zymo Research, Orange, CA, USA). In the bisulfite reaction, the samples were cycled 16 times for 30 sec at 95 °C and 1 h at 50 °C. Then, bisulfite-converted DNA was used for methylation microarrays.

### Gene expression and methylation profiling

mRNA expression profiling was performed by Human Genome U133 plus 2.0 arrays (Affymetrix, Inc., Santa Clara, CA, USA) based on reverse transcription and probe hybridization. This platform contains 41,789 probes. Gene expression levels were detected by relative fluorescence intensity. The expression array data of this study have been submitted to the Gene Expression Omnibus database (accession number GSE19804).

To identify the DNA methylation status of 27,578 CpG sites, the Illumina Infinium Human Methylation27 beadchip (Illumina, San Diego, CA, USA) was used. The accession number for the methylation array data set in the Gene Expression Omnibus database is GSE49996. The methylation levels (beta values) of a given gene were determined by ratio of the methylated probe intensity to the overall probe intensity of that gene. Methylation beta values were then converted to an M-value through a logistic transformation and expressed as the log_2_ ratio of the intensities of methylated probe versus unmethylated probe. The M-value for the i^th^ interrogated CpG site is defined as:


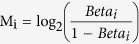


Furthermore, two-dimensional principal component analyses were used for a visual representation of differential expression patterns between tumor and normal samples. Next, hierarchical cluster analysis using Pearson correlation distances was executed to group the probes with similar expression and methylation profiles. Then, differentially expressed and methylated probes were analyzed by paired t tests (*P* ≤ 10^−6^). Genes with differential expression between tumor and adjacent normal tissues were further filtered by at least 2-fold changes. A negative correlation coefficient (r < 0) was used to identify a converse relationship between gene expression and methylation status. A stringent correlation coefficient, defined as r ≤ −0.5, was used for selecting genes for further validation in *in vitro* cell models.

### Cell culture

Cancerous lung cells (A549 and H1299) and normal lung cells (BEAS-2B) were cultured in RPMI medium 1640 (GIBCO, Carlsbad, CA, USA) with 10% fetal bovine serum (FBS; Biological Industries, Beit-Haemek, Israel) and 1% antibiotics, including puromycin and streptomycin (Biological Industries, Beit-Haemek, Israel), at 37 °C in a humidified atmosphere with 5% CO_2_. To validate the role of methylation in regulating the expression of target genes, A549, H1299, and BEAS-2B cells were seeded on a 6-well plate. After 24 h, the cell lines were treated with 5 and 10 μM of 5-aza-2′-deoxycytidine (5-aza) (Sigma Chemical Company, St. Louis, MO, USA). Expression values of target genes were analyzed 3 days after treatment.

### Quantitative reverse transcription PCR

The quality and quantity of the RNA were measured by NanoDrop™ 2000 (Thermo Scientific™, USA). One μg of total RNA from each cell line was reverse transcribed by the High Capacity cDNA Reverse Transcription Kit (Life Technologies, Gaithersburg, MD, USA). The final cDNA products were used as the templates for subsequent real time PCR (RT-PCR). RT-PCR was performed with SYBR Green (Roche, Germany) on an ABI 7900 system (Life Technologies, Gaithersburg, MD, USA) according to standard protocols. The primers used in this study are listed in Table S1. All individual experiments were carried out in triplicate, and data were normalized using *GAPDH* as the loading control. The statistical significance of gene expression in different samples was identified by the t-test calculator in GraphPad Prism 5 (GraphPad Software, Inc., CA, USA).

### Pyrosequencing assay

To quantify the methylation levels of multiple CpG sites in the 5′ untranslated region of *STXBP6*, a predesigned PyroMark CpG Assay was used (Hs_*STXBP6*_01_PM PyroMark CpG assay, PM00057414, Qiagen, Venlo, Netherlands). Specific CpG sites including Chr14: 25518720, 25518735, 25518737, 25518743, and 25518748 were examined.

### Overexpression of *STXBP6* in lung cancer cells

*STXBP6* was overexpressed in A549 and H1299 cells to evaluate its functional significance. Full-length *STXBP6* cDNA with a C-terminal Myc-DDK tag was inserted into the pCMV6-Entry mammalian vector (OriGene Technologies, Rockville, MD, USA). The pCMV6-Entry-Myc-*STXBP6* vector and an empty vector were transiently transfected into A549 and H1299 cell lines using TransIT-2020 transfection reagent (MirusBio, Madison, WI, USA) according to the manufacturer’s instruction. All sequences, including *STXBP6* in the vector, were verified by Sanger sequencing (the first core laboratory, College of Medicine, National Taiwan University). mRNA levels were quantified by quantitative RT-PCR using *STXBP6*-specific primers (F-5′-GTCTATACTTACTGCCAGCG-3′ and R-5′-GTTAAATGCCTTGATGGCCTC-3′), and protein levels were examined by western blotting.

### Western blot

Total cell lysates were prepared and proteins were separated by 10% sodium dodecyl sulfate polyacrylamide gel electrophoresis (SDS-PAGE). Proteins in the gel were then electrotransferred to polyvinylidene difluoride membranes (Bio-Rad Laboratories, Hercules, CA, USA). The membranes were blocked with 5% milk and were incubated with monoclonal anti-FLAG antibody (Sigma-Aldrich, St. Louis, MO, USA) or anti-GAPDH antibody (Sigma-Aldrich, St. Louis, MO, USA) overnight. After washing, the bound primary antibodies on the membranes were incubated with horseradish peroxidase-conjugated anti-rabbit IgG or rabbit anti-mouse IgG (GeneTex, Irvine, CA, USA). Finally, the blots were developed with a chemiluminescent western blotting system (Millipore, Billerica, MA, USA).

### Cell proliferation assay

A549 and H1299 cells were seeded into 96-well plates in triplicate and incubated for 12 h at 37 °C in a CO_2_ incubator. Next, all cells in 96-well plates were divided into groups and transfected with *STXBP6* plasmid or mock vectors. At different time points (24, 48, and 72 h) of transfection, proliferative activity was determined by the 3-(4,5-dimethylthiazol-2-yl)-2,5-diphenyltetrazolium bromide (EMD Biosciences, La Jolla, CA, USA) assay using a microtiter plate reader (BioTek, Winooski, VT, USA) at 570 nm. The absorbance of A549 and H1299 cells was measured.

### Colony formation assay

Cells were seeded in 6-well plates and incubated overnight. The adherent cells were transfected with *STXBP6* plasmid or mock vector. After two weeks of incubation, cells were fixed using 3:1 methanol-acetic acid and stained using 0.1% crystal violet. Finally, the dried plates were used for image acquisition with a digital camera.

### Cell migration assay

Migration assays were carried out using 24-well transwell units (Corning, NY, USA). The upper chamber of each transwell unit was loaded with 4 × 10^4^ cells/well in 0.2 mL serum-free RPMI medium and the lower chambers contained 0.6 mL of RPMI with 10% FBS as chemoattractant. Cells were then incubated for 24 h at 37 °C. Then a methanol-acetic acid (3:1) mixture was added into the lower chambers to fix the cells for 20 min at room temperature, followed by staining with 0.1% crystal violet for another 20 min. Cells on the upper side of the membrane surface were removed by scraping with a cotton swab, and the cells that passed through the filter were destained using 10% acetic acid. The absorbance was measured at 570 nm with an ELISA reader (BioTek, Winooski, VT, USA). Images of the bottom surface of the transwell migration chambers were captured at 10X magnification before destaining.

### Apoptosis assay

In order to perform the annexin V-FITC and propidium iodide (PI) double staining assay, cells were trypsinized, washed with phosphate-buffered saline (PBS), and resuspended in 500 μL of 1X binding buffer (Becton Dickinson, NJ, USA). Thereafter, cells were stained using 10 μL of Annexin V (5 μL) and PI (5 μL) mix (Becton Dickinson, NJ, USA) for 15 min. The suspension was passed through a nylon mesh filter and analyzed using a Beckman Coulter FC500 (Beckman, Brea, CA, USA) and CXP analysis software.

### Cell cycle analysis

Initially, cells were trypsinized, washed with PBS, and fixed with cold 100% ethanol at −20 °C overnight. Thereafter, cells were washed twice and resuspended in PBS containing 20 μg/mL PI (Life Technologies, NY, USA), 0.1% triton-X-100 (Sigma, St. Louis, MO, United States), and 100 μg/mL RNase A (Sigma, St. Louis, MO, United States) for 30 min. The suspension was passed through a nylon mesh filter and analyzed using a Beckman Coulter FC500 (Beckman, Brea, CA, USA) and CXP analysis software.

### Survival analysis

The gene expression signatures from GSE68465^35^ and GSE13213^36^ were used to elucidate the prognostic roles of *STXBP6* in lung adenocarcinoma patients. Patients were categorized as “*STXBP6* High” if their RNA expression levels of *STXBP6* were higher than the median expression in all samples, and as “*STXBP6* Low” if their RNA expression levels of *STXBP6* were lower than the median expression in all samples. The association between gene expression and overall survival (up to 100 months) of lung adenocarcinoma patients was examined using Kaplan-Meier survival analysis. The statistical significance of the relationship between gene expression and survival was examined by a log-rank test.

### Statistical analysis

Data were expressed as the means ± SDs from at least three independent experiments. The statistical significance of gene expression in different samples was identified by a t-test calculator in GraphPad Prism 5 (GraphPad Software, Inc., CA, USA). *P*-values less than 0.05 were considered significant.

## Additional Information

**How to cite this article**: Lenka, G. *et al*. Identification of Methylation-Driven, Differentially Expressed *STXBP6* as a Novel Biomarker in Lung Adenocarcinoma. *Sci. Rep.* **7**, 42573; doi: 10.1038/srep42573 (2017).

**Publisher's note:** Springer Nature remains neutral with regard to jurisdictional claims in published maps and institutional affiliations.

## Supplementary Material

Supplementary Materials

Supplementary Dataset 1

Supplementary Dataset 2

## Figures and Tables

**Table 1:** Genes with negative correlation between gene expression and methylation.

Gene	Exp. Probe ID^a^	Methylation Probe ID^b^	CpG Target No.	Corr.^c^	Exp. Diff. (log_2_)^d^	*P*-value for Exp.^e^	Beta Diff. (T vs N)	Tumor Beta value	Normal Beta value	Methyla-tion Diff. (M value)^f^	*P*-value for Methyla-tion^g^
*AQP1*	209047_at	4600	cg04551925	−0.63	−1.83	2.10E-08	0.16	0.73	0.57	1.03	1.52E-07
*HABP2*	206010_at	26621	cg26656452	−0.61	1.89	3.91E-07	−0.23	0.58	0.81	−1.76	1.33E-08
*SPDEF*	220192_x_at	17272	cg17240454	−0.59	0.81	1.88E-08	−0.19	0.55	0.74	−1.12	2.64E-08
*EHF*	232361_s_at	18421	cg18414381	−0.59	1.29	7.14E-07	−0.17	0.28	0.45	−0.93	1.52E-09
*HMGN1*	200944_s_at	18826	cg18829411	−0.57	0.58	1.60E-08	−0.16	0.31	0.47	−1.01	4.89E-09
*SLC22A18*	204981_at	16913	cg16873863	−0.57	1.07	1.79E-07	−0.23	0.40	0.63	−1.35	1.90E-09
*ARHGEF19*	226857_at	18670	cg18669381	−0.56	1.20	7.74E-09	−0.11	0.29	0.40	−0.69	2.32E-07
*IL11RA*	204773_at	3676	cg03662459	−0.56	−0.74	8.98E-08	0.09	0.68	0.59	0.59	2.00E-07
*GSTM5*	205752_s_at	5031	cg04987894	−0.55	−0.69	1.57E-07	0.21	0.52	0.31	1.04	3.51E-11
*NIPSNAP1*	201709_s_at	13745	cg13797031	−0.55	0.70	2.14E-08	−0.26	0.49	0.75	−1.79	3.35E-09
*MESP1*	224476_s_at	1105	cg01091565	−0.54	1.24	9.77E-07	−0.18	0.62	0.80	−1.28	1.72E-08
*EHF*	232361_s_at	13011	cg13084525	−0.54	1.29	7.14E-07	−0.08	0.20	0.28	−0.71	1.36E-08
*CP*	228143_at	17465	cg17439694	−0.53	2.57	2.65E-07	−0.13	0.54	0.67	−0.91	2.26E-07
*SPDEF*	220192_x_at	7679	cg07705908	−0.53	0.81	1.88E-08	−0.21	0.36	0.57	−1.13	1.90E-08
*HDHD3*	221256_s_at	23967	cg24012708	−0.53	0.59	3.26E-07	−0.15	0.44	0.59	−0.95	1.53E-07
*DCC*	238914_at	4292	cg04272086	−0.52	−0.98	2.90E-09	0.13	0.20	0.07	1.48	1.21E-10
*STXBP6*	220994_s_at	6937	cg06948294	−0.52	−2.07	8.79E-12	0.11	0.15	0.04	1.62	1.68E-08
*COX7A1*	204570_at	24277	cg24335895	−0.52	−1.27	7.66E-10	0.22	0.52	0.30	1.44	2.71E-11
*SPDEF*	213441_x_at	17272	cg17240454	−0.52	0.53	5.98E-08	−0.19	0.55	0.74	−1.12	2.64E-08
*RHOJ*	243481_at	18774	cg18771300	−0.52	−1.25	1.19E-10	0.21	0.52	0.31	1.05	1.20E-07
*CP*	1558034_s_at	17465	cg17439694	−0.51	2.52	3.28E-09	−0.13	0.54	0.67	−0.91	2.26E-07
*PECAM1*	208981_at	3928	cg03886110	−0.50	−1.49	1.19E-10	0.20	0.72	0.52	1.07	2.39E-11

^a^Probes used for the gene expression profiling.

^b^Probes used for the methylation analysis.

^c^Correlation between gene expression and methylation status.

^d^Differential median values of gene in tumor/normal tissue samples (log_2_ ratio).

^e^Statistical significance of gene expression by paired t test.

^f^Differential median values of methylated probes versus unmethylated probes (M value).

^g^Statistical significance of methylation examined by paired-t test.

**Table 2:** Expression values and methylation status of *STXBP6* in lung adenocarcinoma among public data set.

Data Type	GSE No. (^reference^)	Sample Size	Correlation^a^	Expression Difference (log_2_)^b^	*P*-value for Expression^c^	Methylation Difference (M value)^d^	*P*-value for Methylation^e^
Expression							
	GSE19188^47^	110		−4.22**	3.72E-20		
	GSE7670^49^	56		−1.37**	1.40E-13		
	GSE31210^48^	246		−3.77**	2.47E-12		
	GSE10072^50^	107		−1.52**	1.06E-26		
Methylation							
	GSE19034	60				2.24*	2.48E-03
	GSE32866^44^	50				2.04**	3.58E-08
Expression & Methylation							
	GSE19804/GSE49996	120/64	−0.52	−4.38**	9.57E-22	1.73**	8.61E-08
	GSE62949/GSE62948	56/56	−0.60	−0.24**	3.84E-11	1.08**	5.98E-08
	GSE32863/GSE32861(44)	114/118	−0.68	−1.83**	9.29E-22	2.54**	2.49E-18
	GSE63459/GSE63384^51^	62/70	−0.53	−1.79*	4.20E-02	1.34**	2.21E-08

^a^Correlation between gene expression and methylation status.

^b^Differential median values of gene in tumor/normal tissue samples (log2 ratio).

^c^Statistical significance of gene expression by t test or paired-t test.

^d^Differential median values of methylated probes versus unmethylated probes (M value).

^e^Statistical significance of methylation examined by paired-t test.

^*^P < 1.00E-2; **P < 1.00E-07.
