# Supplementary material for: Identification of Methylation-Driven, Differentially Expressed STXBP6 as a Novel Biomarker in Lung Adenocarcinoma
Source: Sci Rep. 2017 Feb 15;7:42573. doi: 10.1038/srep42573 (PMC5309775; doi:10.1038/srep42573)
Supplement: Supplementary Materials [file srep42573-s1.doc]

Identification of Methylation-Driven, Differentially Expressed *STXBP6* as a Novel Biomarker in Lung Adenocarcinoma

Govinda Lenka, Mong-Hsun Tsai, Hsin-Chieh Lin, Jen-Hao Hsiao, Yi-Ching Lee, Tzu-Pin Lu, Jang-Ming Lee, Chung-Ping Hsu, Liang-Chuan Lai^*^ and Eric Y. Chuang^*^

**Table S1.** List of primers used for validation of selected genes by qRT-PCR analysis.

| **Gene Symbol** | **Gene Description** | **Primers (Forward/Reverse)** |
| --- | --- | --- |
|  |  |  |
| *GSTM5* | Glutathione S-transferase mu 5 | 5'-TCCTTGCCTATGATGTCCTTGA-3' |
|  |  | 5'-AGGAAGGCGTCCAAGCACTT-3' |
| *IL11RA* | Interleukin 11 receptor, alpha | 5'-GCCAAGCAGCCGACTATGAG-3' |
|  |  | 5'-AGGTAGCGGGTGGGTAAACC-3' |
| *PECAM1* | Platelet/endothelial cell adhesion molecule 1 | 5'-AATGATCCTGCGGTATTCAAAGAC-3' |
|  |  | 5'-ATTATCTGCAACACACTGGTATTCG-3' |
| *RHOJ* | Ras homolog family member J | 5'-CAACACTTGCTCGGACTGTATGAC-3' |
|  |  | 5'-AGTGGCCTCAGCTGGTTGTAG-3' |
| *STXBP6* | Syntaxin binding protein 6 (amisyn) | 5'-GCCAGCACAGCGTCAGAA-3' |
|  |  | 5'-TGGCAGGTATGGTGGAGGAT-3' |
| *GAPDH* | Glyceraldehyde-3-phosphate dehydrogenase | 5'-TGCACCACCAACTGCTTAG-3' |
|  |  | 5'-GATGCAGGGATGATGTTC-3' |

**Table S2.** Clinical characteristic of patients in this study.

| **Characteristic** | **Sample size, n (%)** | **Exp. Diff. (log2)^a^** | ***P*-value for Exp.^b^** | **Methylation Diff.**  **(M value)^c^** | ***P*-value for Methylation^d^** | |  |
| --- | --- | --- | --- | --- | --- | --- | --- |
| Stage |  |  |  |  | |  | |
| I+II | 24 (75) | -0.83 ± 1.12 | 0.44 | 1.20 ± 0.92 | | 0.20 | |
| III+IV | 8 (25) | -0.77 ± 0.92 |  | 1.58 ± 1.52 | |  | |
| Age^#^ |  |  |  |  | |  | |
| ≧62 | 15 (47) | -0.73 ± 1.16 | 0.34 | 1.33 ± 1.20 | | 0.43 | |
| <62 | 17 (53) | -0.89 ± 1.00 |  | 1.26 ± 1.02 | |  | |

^#^Range (39-79 yr, mean = 62 yr)

**^a^** Differential median values of gene in tumor/normal tissue samples (log_2_ ratio).

**^b^** Statistical significance of gene expression by t test.

**^c^** Differential median values of methylated probes versus unmethylated probes (M value).

**^d^** Statistical significance of methylation examined by t test.


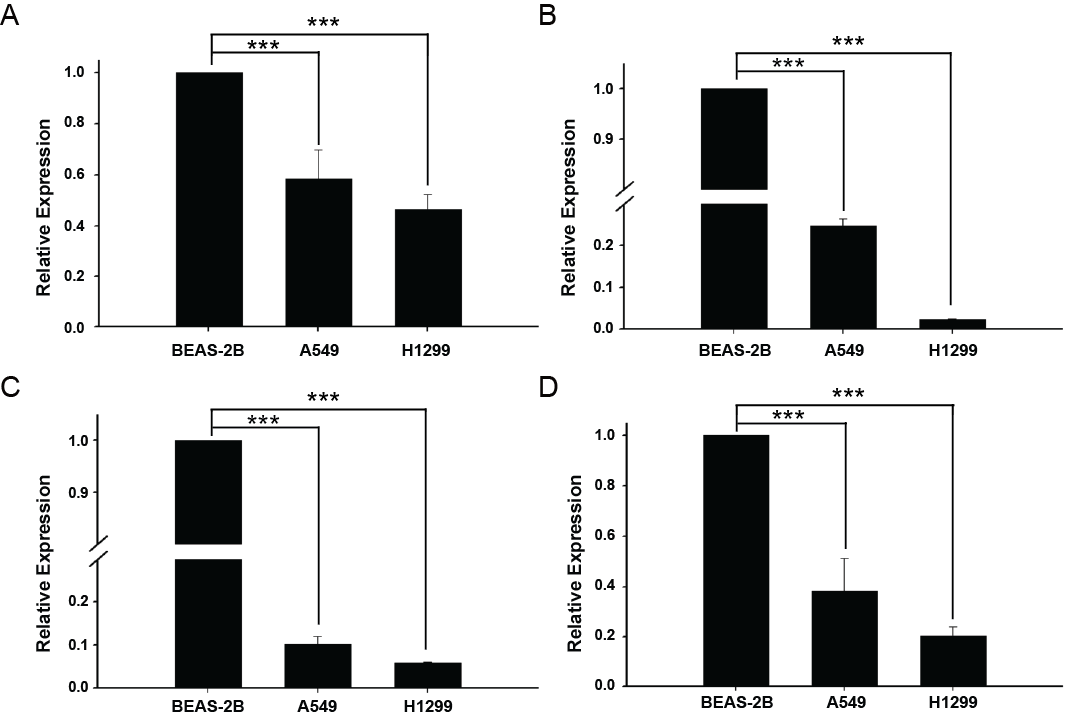


**Figure S1. Validation of negatively correlated genes with lower endogenous expression levels in lung cancer cells.** mRNAs were extracted from A549 and H1299 cancer cells and BEAS-2B normal control cells. The endogenous expression values were measured by qRT-PCR for the selected gene targets, including *IL11RA* **(A)**, *GSTM*5 **(B)**, *RHOJ* **(C)** and *PECAM1* **(D)**. Bars represent the means ± SDs of 3 independent experiments. Data for all genes were normalized against *GAPDH*. Gene expression level in A549 and H1299 cells was normalized to that in BEAS-2B cells. ***, *P* ≤ 0.0001.
